# Successful Treatment of Adenovirus Infection with Brincidofovir in an Immunocompromised Patient after Hematological Stem Cell Transplantation

**DOI:** 10.1155/2020/5981289

**Published:** 2020-01-07

**Authors:** T. Van Genechten, J. van Heerden, T. Bauters, C. Dhooge

**Affiliations:** ^1^Pediatric Haematology Oncology and Stem Cell Transplantation, Ghent University Hospital, Ghent, Belgium; ^2^Pediatric Haematology and Oncology Department, Antwerp University Hospital, Antwerp, Belgium; ^3^Pharmacy Department, Ghent University Hospital, Ghent, Belgium

## Abstract

Immunocompromised patients, including hematopoietic stem cell transplantation (HSCT), HIV, and malnourished patients, are at increased risk for viral infections with high incidences of morbidity and mortality. In HSCT patients, the infection risk is increased until immune reconstitution is re-established. Therapy with standard of care antiviral drugs, for example Cidofovir, is expensive, requires prolonged administration, and has unfavorable toxicity profiles. Our case describes the successful use of Brincidofovir (CMX001), a lipid-conjugate of the nucleotide analog Cidofovir, in a 9-year-old post-HSCT girl with disseminated adenovirus infection. The increased efficacy of Brincidofovir (BCV) against multiple viral infections, limited toxicity, and oral-administered schedule opens options in different resource settings.

## 1. Case Report

**Figure 1:**
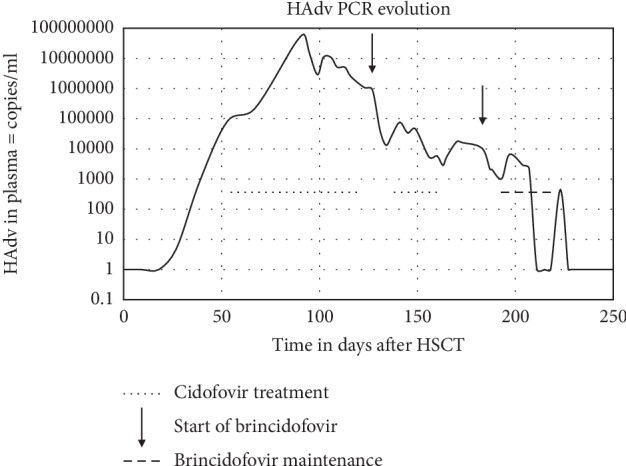
The viral load response during the treatment with Cidofovir and Brincidofovir.

A 9-year-old girl diagnosed with transfusion-dependent refractory cytopenia of childhood (RCC) was treated with a T-cell-depleted haploidentical transplantation from her mother, followed by a second T-cell-depleted haploidentical transplantation from her father due to graft failure. Post-engraftment, she presented with general malaise, weight loss, and vomiting. Concomitant Epstein–Barr viral (EBV) reactivation, Herpes simplex (HSV) infection, and Human adenovirus (HAdV) were confirmed by plasma polymerase chain reaction (PCR). EBV was successfully treated with rituximab and HSV with acyclovir. However, as the HAdV viral load (VL) increased to 132 × 10^6^ copies/ml, treatment with 5 mg/kg weekly intravenous cidofovir with concomitant hydration only obtained a moderate decrease in the viral load (Figure 1). The patient rapidly developed renal toxicity. After a minimum of 48-hour dose-to-dose washout interval, after administration of the last dose of intravenous Cidofovir, the first dose of BCV suspension was started. BCV was administered at an oral dose of 2 mg/kg, twice weekly. This successfully suppressed the VL with significant clinical improvement. Cholestasis developed one week after the start of BCV treatment. As there were no other concomitant drugs that could have caused cholestasis, BCV treatment was stopped and Cidofovir was restarted. However, as HAdV reactivated, BCV was restarted after resolution of the cholestasis, clearing the VL in the absence of immune reconstitution.

## 2. Discussion

HAdV is a non-enveloped double-stranded DNA virus with over 80 known virus types, divided into seven species [1–3]. Maternal antibody protection prevents infections before 6 months of age, whereafter endemicity is established in over 80% of children by the age of 6 years [4]. The incidence does not vary between different countries, but spread, morbidity, and mortality are increased in regions with limited sanitation such as low- and middle-income countries (LMIC) as transmission occurs via droplet, feco-oral, and direct spread [5].

After primary infection the virus remains latent in the lymphoreticular system. In immunocompetent individuals persistent shedding is present, but in immunocompromised individuals, reactivation increases morbidity and mortality rates [2, 4].

Our patient was both T- and B-cell-depleted due to the double haploidentical HSCT, conditioning regimens with antithymocyte globulin and rituximab treatment. Without HAdV specific CD4+ helper, CD8+ cytotoxic T cells and inadequate clearance, the HAdV caused a symptomatic viraemia [6, 7]. Other immunocompromised patients (e.g., severe combined immunodeficiency or HIV-patients) might as well benefit from easily accessible drugs with a reasonably safe toxic profile [8].

**Figure 2:**
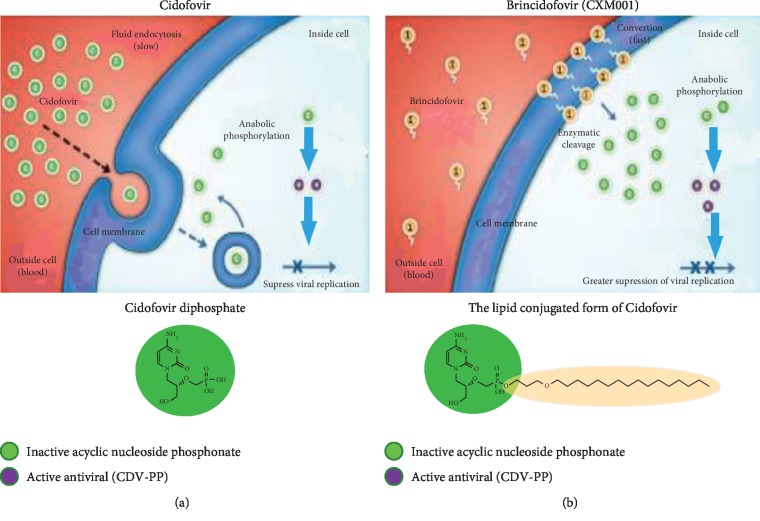
Pharmacokinetics and drug composition of Cidofovir and Brincidofovir. (a) The activated antiviral, cidofovir diphosphate, leads to chain termination as it is incorporated into the viral DNA. (b) In Brincidofovir, the lipid conjugated form of Cidofovir, intracellular uptake is increased leading to a more than 100-fold increase in intracellular concentration of active cidofovir.

Treatment of HAdV infection in immunocompromised patients is ineffective without immune reconstitution [6, 7]. Cidofovir requires regular intravenous dosing but only achieves low in vivo activity due to low intracellular levels (see Figure 2(a)), and therefore poor results [9]. This might obtain viral control but hardly ever viral clearance. Cidofovir is most effective as prophylaxis with increasing titres on stool samples, plasma PCR's or early onset disease [10]. The side effect profile includes nausea, vomiting, myelosuppression and severe renal tubulopathies [8]. During treatment the direct and indirect costs increase due to in-hospital toxicity monitoring and mandatory adequate hydration.

Brincidofovir, the lipid-conjugate of the nucleotide analog Cidofovir, can achieve over 100-fold higher intracellular levels compared with Cidofovir (see Figure 2(b)). The low plasma levels promote less toxicity, and nephrotoxicity is avoided as there is no binding to anion transporters in the kidney [8, 11]. BCV has a twice weekly oral dosing, low toxicity, and broad spectrum antiviral activity, including CMV, EBV, acyclovir-resistant HSV, and BK-virus, even without immune reconstitution [11].

Adoptive immunotherapy by means of donor leukocyte infusion has been used, curing HAdV infection, but carries the risk of developing graft versus host reactions. Therapies such as modified HAdV-specific T-cells are expensive and time consuming with complex administration procedures [12].

In LMIC, high viral infection susceptibilities in patients with HIV, malnutrition, chronic diseases, malignancies, as well as increasing organ transplantation, family-related donor and haploidentical HSCT, resource sensitive alternatives are essential. Brincidofovir could fill in this gap, provided its availability is guaranteed.

## 3. Conclusion

BCV can successfully treat HAdV infection in post-transplant immunocompromised children. Although direct drug costs have not been determined, indirect cost savings on hospitalization, staffing, screening, and procedures in multiple disease profiles makes BCV a promising drug for resource-limited settings.
